# Glochodpurnoid B from *Glochidion puberum* Induces Endoplasmic Reticulum Stress-Mediated Apoptosis in Colorectal Cancer Cells

**DOI:** 10.3390/molecules28020511

**Published:** 2023-01-04

**Authors:** Yang Tian, Runzhu Fan, Zhao Yin, Yongping Huang, Dong Huang, Fangyu Yuan, Aiping Yin, Guihua Tang, Rong Pu, Sheng Yin

**Affiliations:** 1 School of Pharmaceutical Sciences, Sun Yat-sen University, Guangzhou 510006, China; 2 Department of Geratology, The Affiliated Hospital of Yunnan University, The Second People’s Hospital of Yunnan Province, Kunming 650021, China; 3 School of Life Sciences and Food Engineering, Hanshan Normal University, Chaozhou 521041, China; 4 Department of Clinical Laboratory, The Third People’s Hospital of Dongguan, Dongguan 523326, China

**Keywords:** *Glochidion puberum*, triterpenoid, structural elucidation, apoptosis, endoplasmic reticulum stress

## Abstract

Glochidpurnoids A and B (**1** and **2**), two new coumaroyl or feruloyl oleananes, along with 17 known triterpenoids (**3**–**19**) were obtained from the stems and twigs of *Glochidion puberum*. Their structures were elucidated by extensive spectroscopic data analyses, chemical methods, and single crystal X-ray diffraction. All compounds were screened for cytotoxicity against the colorectal cancer cell line HCT-116, and **2**, **3**, **5**, **6**, **11**, and **17** showed remarkable inhibitory activities (IC_50_: 0.80–2.99 μM), being more active than the positive control 5-fluorouracil (5-FU). The mechanistic study of **2**, the most potent compound, showed that it could induce endoplasmic reticulum (ER) stress-mediated apoptosis and improve the sensitivity of HCT-116 cells to 5-FU.

## 1. Introduction

Colorectal cancer (CRC) is the third most commonly diagnosed and the second deadliest cancer worldwide [1]. According to global cancer statistics, approximately 1.9 million new CRC cases and 935,000 CRC deaths occurred in 2020, and this number is predicted to be increasing annually [2]. In recent years, although some novel therapies, such as targeted therapy, immunotherapy, and gene therapy, have shown certain benefits in CRC treatment, fluoropyrimidine (5-FU)-based chemotherapy still remains the first-line treatment for CRC [3]. Patients with poor performance or low risk of deterioration are recommended 5-FU single therapy. As for 5-FU-resistance patients, multiple-agent regimens, such as 5-FU + oxaliplatin, 5-FU + irinotecan (IRI), are often adopted. Choosing additive agents appears to be similar in efficacy, with only side effects varying [4]. However, inevitable deficiencies, such as systemic toxicity, low response rate, as well as acquired resistance, limit the clinical application of 5-FU [5]. Thus, developing more effective therapeutic drugs with less side effects for CRC is still urgent.

Natural products have been evolutionarily selected to bind to biological macromolecules and, thus, represent a valuable source of “privileged structures” in drug discovery [6]. Such role is especially prominent in anticancer research, with ca. 70% of FDA-approved anticancer drugs being either natural products or their derivatives [7]. In the treatment of CRC, many natural products are currently undergoing different phases of clinical trials, such as andrographolide (phase II), berberine (phase II/III), curcumin (phase I), epigallocatechin gallate (phase II), resveratrol (phase I), and silymarin (phase IV) [8]. These compounds have the potential to exert anti-CRC effects by interfering with the pathways of metastasis, invasion, apoptosis, and angiogenesis. In the past few years, our group has endeavored to discover anticancer agents from Euphorbiaceae plants, and has proven that terpenoids from Euphorbiaceae are a rich source of privileged structures in anticancer drug development [9].

*Glochidion puberum* (L.) Hutch (Euphorbiaceae) is a shrub widely distributed in southwest China [10]. The leaves, stems, roots, and fruits of *G. puberum* are used in traditional Chinese herbal drugs to treat dysentery, diarrhea, influenza, fever, cough, and impaludism [11]. According to literature researches, the chemical components isolated from this genus include triterpenoids, alkaloids, flavonoids, steroids, ligans, phenolic and so on [12]. Especially, some triterpenoids from *Glochidion* possessed antitumor-promoting and cytotoxic activities [13,14]. In our continued efforts toward discovering structurally diverse and bioactive triterpenoids from Euphorbiaceae plants [15,16,17], two new oleanane derivatives, glochidpurnoids A (**1**) and B (**2**), along with 17 known analogues were obtained from the stems and twigs of *G. puberum*. All their cytotoxicity was measured using MTT assay, and six compounds showed pronounced anti-CRC activities against HCT-116 cells. Derivative **2**, with the best activity, was selected for in-depth mechanism exploration. Herein, details of the isolation, structural identification, along with anti-CRC potential of these compounds were reported.

## 2. Results and Discussion

### 2.1. Isolation and Structure Elucidation

**Figure 1:**
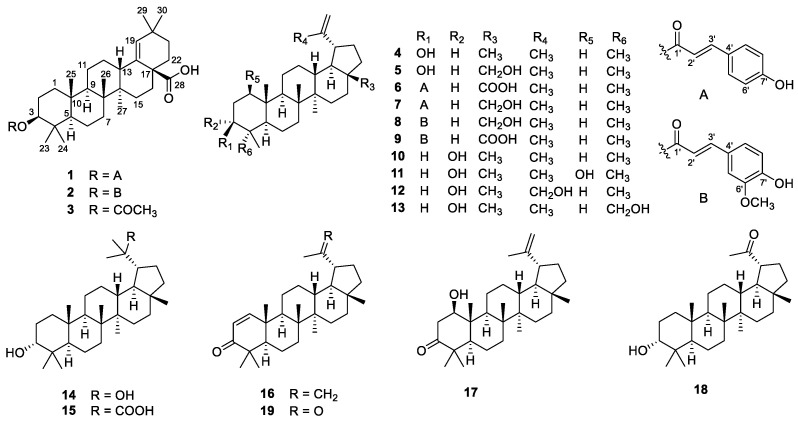
Structures of **1**–**19**.

The air-dried powder of the stems and twigs of *G. puberum* was extracted with 95% EtOH to give a crude extract, which was partitioned between water and EtOAc. The EtOAc extract was separated using multiple column chromatography to obtain triterpenoids **1**–**19** (Figure 1).

**Figure 2:**
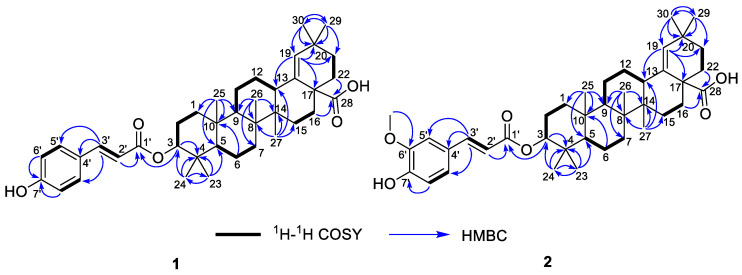
The ^1^H–^1^H COSY and HMBC correlations of **1** and **2**.

**Figure 3:**
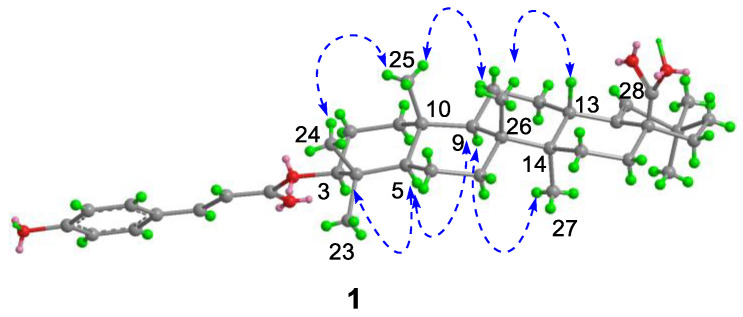
Key NOE correlations of **1**.

**Figure 4:**
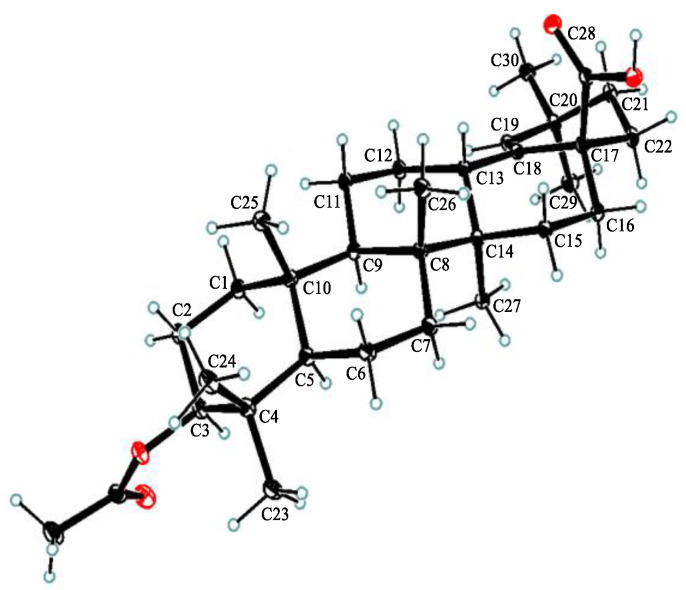
Single-crystal X-ray structure of **3**.

Compound **1**, white powder, possessed the molecular formula C_39_H_54_O_5_ as determined by the HRESIMS ion at *m/z* 625.3853 [M + Na]^+^ (calcd. For C_39_H_54_O_5_Na^+^, 625.3863). The IR absorption bands revealed the presence of carbonyls (1690 and 1605 cm^−1^). The ^1^H NMR data (Table 1) exhibited signals indicative of seven methyl singlets [*δ*_H_ 1.16 (3H, s), 1.10 (3H, s), 1.04 (3H, s), 0.99 (6H, s), 0.97 (3H, s), and 0.82 (3H, s)], one trisubstituted double bond proton [*δ*_H_ 5.33 (1H, s)], a trans-coumaroyl moiety [*δ*_H_ 8.07 (1H, d, *J* = 15.9 Hz), 7.69 (2H, d, *J* = 8.2 Hz), 7.20 (2H, m), and 6.74 (1H, d, *J* = 15.9 Hz)] [18], an oxymethine proton [*δ*_H_ 4.90 (1H, dd, *J* = 11.6, 4.7 Hz)], along with a series of aliphatic multiples. The ^13^C NMR and DEPT135 spectra showed 39 carbon resonances indicative of a trans-coumaroyl group (*δ*_C_ 167.7, 161.8, 145.4, 131.1 × 2, 126.6, 117.3 × 2, and 116.2), one carboxylic carbon (*δ*_C_ 179.4), one trisubstituted double bonds (*δ*_C_ 139.3 and 132.5), ten sp^3^ methylenes, four sp^3^ methines (one oxygenated), seven methyls, and six sp^3^ quaternary carbons. The abovementioned information revealed an oleanane core, morolic acid [19], with a trans-coumaroyl substituent, which was further validated by elucidation of 2D NMR analyses of **1** (Figure 2). In particular, the HMBC cross-peak from H-3 [*δ*_H_ 4.90 (dd, *J* = 11.6, 4.7 Hz)] of the oleanane core to the ester carbonyl (C-1′, *δ*_C_ 167.7) of the coumaroyl moiety located this group at OH-3. The relative configuration of **1**, regarding the oleanane part, was established via interpretation of NOESY data (Figure 3) and by comparing its NMR date with those of morolic acid. As shown in Figure 3, the strong NOE interactions of H-3/H-5 and Me-24/Me-25 disclosed that these methyls or protons were axially oriented on the chair conformational A-ring. Me-24 and Me-25 were arbitrarily assigned β-orientations, and thus H-3 and H-5 were α-oriented. The NOE interactions of H-9/H-5 and Me-27 implied the α-orientations of these protons and methyl group. Correspondingly, the NOE interactions of Me-26/Me-25 and H-13 suggested that H-13 and Me-26 were β-orientation. Finally, the absolute configuration of **1** was determined by comparing the alkaline hydrolysis products with those of **3**, an acetylated derivative of morolic acid also isolated in the current research. Both compounds generated the same morolic acid product. As the absolute configuration of **3** was assigned as 3S,5R,8R,9R,10R,13S,14R,17S by using single crystal X-ray diffraction analysis [Flack parameter = −0.01 (6)] (Figure 4 in the current study), the structure of **1** with the same absolute configuration was established as depicted and named glochidpurnoid A.

Compound **2** exhibited a molecular formula of C_40_H_56_O_6_ based on HRESIMS ion peak at *m/z* 655.3972 [M + Na]^+^ (calcd. For C_40_H_56_O_6_Na^+^, 655.3969). The 1D NMR data of **2** showed high similarity to those of **1**, with the only difference being the presence of an additional aromatic methoxyl signal (*δ*_H_ 3.93, *δ*_C_ 55.9) in **2**, suggesting that **2** was a methoxylated derivative of **1**. The location of methoxyl was authenticated by the HMBC correlation from 6′-OCH_3_ (*δ*_H_ 3.93) to the C-6′ (*δ*_C_ 146.7). Derivative **2** had the same relative configuration as that of **1** based on their similar NOE correlations. Similarly, the alkaline hydrolysis of **2** produced the same morolic acid product as those of **1** and **3**, suggesting that they possessed the same absolute configuration. Hence, the structure of **2** was determined as shown and named glochidpurnoid B.

The known compounds, morolic acid acetate (**3**) [20], lupeol (**4**) [21], betulin (**5**) [22], 3β-O-trans-coumaroylbetulinic acid (**6**) [18], 3β-O-trans-coumaroylbetulin (**7**) [23], 3β-O-trans-feruloylbetulin (**8**) [24], 3β-O-trans-feruloylbetulinic acid (**9**) [25], epilupeol (**10**) [26], glochidiol (**11**) [14], lup-20(30)-ene-3α,29-diol (**12**) [27], lup- 20(29)-ene-3α,23-diol (**13**) [28], 3α,20-lupandiol (**14**) [29], (20S)-3α-hydroxylupan-29-oic acid (**15**) [30], glochidone (**16**) [31], glochidonol (**17**) [32], obibanmol I (**18**) [33], and 29-nor-lup-1-ene-3,20-dione (**19**) [34] were determined based on their identical NMR data to those in the literature.

### 2.2. ***2*** Inhibited Cell Proliferation in Colorectal Cancer (CRC) Cell Line HCT-116

**Figure 5:**
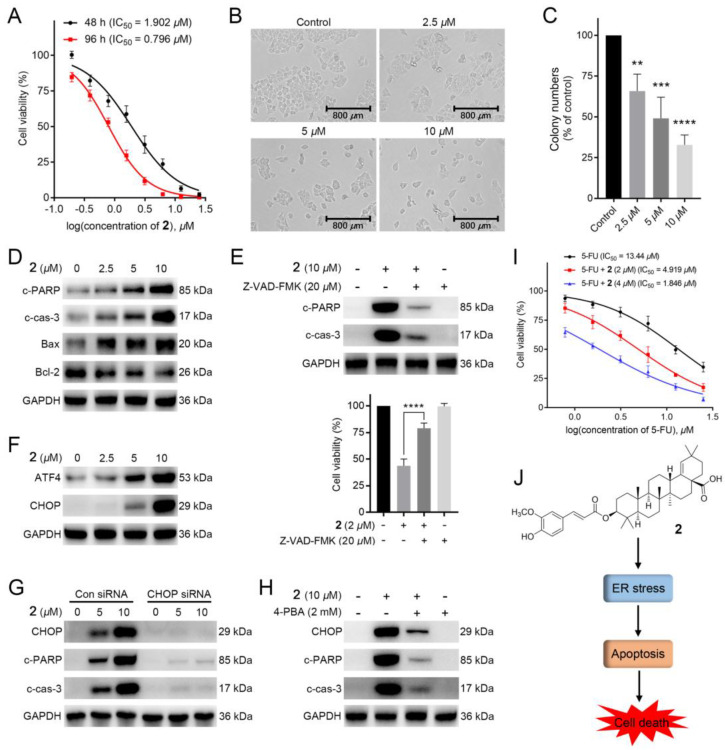
**2** exerted potent cytotoxic activity in colorectal cancer cells: (**A**) The anti-proliferative effects of **2** on HCT-116 cells. MTT assay was applied to determine the cell viability of HCT-116 cells treated with several concentrations of **2** for 48 and 96 h; (**B**) The cell morphology of HCT-116 cells with the administration of several concentrations (0, 2.5, 5, and 10 μM) of **2** for 12 h; (**C**) The colony assay of HCT-116 cells with the administration of different concentrations (0, 2.5, 5, and 10 μM) of **2**; (**D**) Western blotting of the expressions of c-PARP, c-cas-3, Bax and Bcl-2 in HCT-116 cells. Cells were treated with or without several concentrations (2.5, 5, and 10 μM) of **2** for 12 h; (**E**) Effects of apoptosis on **2**-induced cell death in HCT-116 cells. The expression levels of c-PARP and c-cas-3 in HCT-116 cells treated with **2** (10 μM), Z-VAD-FMK (20 μM), or both were measured by western blotting. MTT assay was conducted to determine the cell viability of HCT-116 cells with administration of Z-VAD-FMK (20 μM) followed by **2** (2 μM) for 12 h; (**F**) Western blotting analysis of key transcription factors (ATF4 and CHOP) in HCT-116 cells. Cells were added into several concentrations (0, 2.5, 5, and 10 μM) of **2** for 12 h; (**G**) Western blotting analysis of the effect of CHOP knockdown on **2**-induced protein changes. Cells with or without CHOP knockdown were added into **2** (5 and 10 μM) for 12h, respectively; (**H**) Western blot analysis of the effect of 4-PBA on **2**-induced proteins changes in HCT-116 cells. Cells with the pre-treatment of 4-PBA (2000 U/mL) were treated with **2** (10 μM) for 12 h; (**I**) The anti-proliferative effects of **2** on the cytotoxicity of 5-FU in HCT-116 cells. Cells were treated with several doses of 5-FU with or without administration of **2** (2 and 4 μM) for 48 h. Cell viability was tested using MTT assay; (**J**) Schematic illustration of the underlying mechanism of anticancer activity of **2** [*n* = 3, ** *p* < 0.01, *** *p* < 0.001 and **** *p* < 0.0001 compared to the control group].

**1**–**19** were tested for their cytotoxicity against HCT-116 cells (5-FU as the positive drug). To our surprise, compounds **2**, **3**, **5**, **6**, **11**, and **17** exhibited remarkable inhibitory activities (IC_50_: 0.80–2.99 μM), being more potent than that of the 5-FU (Table 2). Among them, **2** showed the most potent activity with IC_50_ = 0.80 ± 0.05 μM, and its activity was further confirmed by colony formation assay and time/dose-dependent assays. As illustrated in Figure 5A,B, the cell viability and morphology of HCT-116 cells were dose-dependently and time-dependently deteriorated with the treatment of **2** at 48 or 96 h. Meanwhile, the colony numbers of HCT-116 cells with the administration of **2** in 12-day were dose-dependently decreased, suggesting that **2** could repress the proliferation of HCT-116 cells (Figure 5C).

### 2.3. ***2*** Induced Apoptosis in HCT-116 Cells

Apoptosis is the cell’s natural mechanism for programed cell death. While the apoptotic pathway in cancer cells is typically inhibited, allowing cancer cells to survive longer for the accumulation of mutations which can increase invasiveness during tumor progression, stimulate angiogenesis, deregulate cell proliferation and interfere with differentiation [35]. Thus, apoptosis is considered as a promising target for anticancer therapy. Additionally, previous studies reported that lots of plant-derived compounds exhibit anticancer activity through activating the apoptotic pathway. To explore whether **2** could induce apoptosis, we adapted the western blotting to measure the expressions of apoptosis-related proteins cleaved PARP (c-PARP) and cleaved caspase-3 (c-cas-3), pro-apoptotic protein Bax, and anti-apoptotic protein Bcl-2. As displayed in Figure 5D, the expressions of c-PARP, c-cas-3, and Bax were dose-dependently increased, while Bcl-2 expression was downregulated with the treatment of **2** in HCT-116 cells, indicating that **2** could trigger apoptosis. To verify this, we further conducted apoptosis-related characteristic experiments. As shown in Figure 5E, Z-VAD-FMK, an apoptosis inhibitor, could dose-dependently attenuate **2**-induced cell death and downregulate the expressions of apoptosis-related proteins (c-PARP and c-cas-3). These results suggested that **2** caused HCT-116 cells death via apoptosis.

### 2.4. ***2*** Stimulated Endoplasmic Reticulum (ER) Stress-Mediated Apoptosis in HCT-116 Cells

A previous study found that pentacyclic triterpenoids could induce endoplasmic reticulum stress, which participates in cell apoptosis in most tumors [36]. We then investigated whether **2** induced apoptosis via ER stress signaling. As shown in Figure 5F, the expressions of ATF4 and CHOP, two marker genes of ER stress, were improved with the addition of **2**, suggesting that **2** could induce ER stress in HCT-116 cells. Furthermore, knockdown of CHOP by siRNAs downregulated the expressions of c-PARP and c-cas-3 (Figure 5G). Meanwhile, **2**-induced increased expressions of CHOP and apoptosis-related proteins in HCT-116 cells could be decreased with the treatment of the ER stress inhibitor 4-PBA (2 mM). (Figure 5H). Taken together, **2**-induced apoptosis in HCT-116 cells was related to ER stress.

### 2.5. ***2*** Potentiated the Antitumor Activity of 5-FU in HCT-116 Cells

As 5-FU, the first-line drug for CRC treatment, performs its efficacy via inhibiting DNA biosynthesis [9], the mechanism of which is different from that of **2**, we further explored whether the combination of 5-FU and **2** could improve the therapeutic activity. As shown in Figure 5I, **2** dose-dependently increased the cytotoxic activity of 5-FU in HCT-116 cells, with 7 times improvement under the administration of 4 μM of **2**. The above result indicated that **2** might possess a synergistic effect with 5-FU in HCT-116 cells, making **2** a promising agent in future combination therap.

## 3. Materials and Methods

### 3.1. Plant Material

The stems and twigs of *Glochidion puberum* (L.) Hutch were collected in June 2020 from Hengyang City, Hunan Province, China, and measured by Prof. Guihua Tang, one of the authors (Tang, G.H.) in our current research. A voucher specimen (accession number: GB2020-1) has been deposited at the School of Pharmaceutical Sciences, Sun Yat-sen University, Guangzhou, China.

### 3.2. General Experimental Procedures

Details of chemical procedures and instruments were displayed in Supplementary Materials.

### 3.3. Extraction and Isolation

The air-dried powder of the stems and twigs of *G. puberum* (13 kg) was extracted with 95% EtOH (3 × 55 L) at room temperature. The EtOH extract (819 g) was suspended in H_2_O (1 L) and partitioned with EtOAc (3 × 2.5 L) to give EtOAc portion (185 g), which was separated by macroporous resin column (MeOH/H_2_OH, 35% → 95%) to afford four fractions (Frs.1–4). Fr.3 (40 g) was purified by silica gel CC eluted with a PE/EtOAc gradient (0:1 → 1:1) to give nine subfractions (Frs. 3a−3i), and **16** (15 mg) was finally separated from Fr.3a (2.4 g) by using silica gel CC (PE/acetone, 2000:1 → 1000:1). Meanwhile, **4** (30 mg) and **10** (10 mg) were purified from Fr.3c (5.6 g) using silica gel CC (PE/EtOAc, 500:1 → 100:1). Separation of the Fr.3e (4.6 g) was conducted using silica gel CC (PE/EtOAc, 100:1 → 10:1) to yield **3** (17 mg), **17** (10 mg) and **18** (11 mg). Fr.3f (2.9 g) was separated by silica gel CC (PE/EtOAc, 20:1 → 10:1) to afford Frs.3f1–3f3. Of them, **5** (23 mg), **13** (17 mg), and **14** (10 mg) were purified from Fr.3f1 (1.5 g) by a Rp-C_18_ column eluted with MeOH/H_2_O (5:5 → 10:0), and Fr.3f2 (1.2 g) was obtained using HPLC (CH_3_CN/H_2_O, 90% → 95%, 3 mL/min) to afford **6** (8 mg, *t*_R_ 21 min), **7** (24 mg, *t*_R_ 23 min), **8** (29 mg, *t*_R_ 30 min), **9** (8 mg, *t*_R_ 32 min), as well as **1** (5 mg, *t*_R_ 19 min) and **2** (30 mg, *t*_R_ 20 min). Furthermore, **15** (6 mg) was purified from Fr.3f3 (30 mg) using Sephadex LH-20 eluted with MeOH. In addition, **11** (8 mg) and **12** (7 mg) were yielded from Fr.3g (1.7 g) by using silica gel CC (PE/EtOAc_,_ 10:1 → 5:1). The purification of Fr.3h (3.9 g) was conducted using Sephadex LH-20 eluted with MeOH to produce **19** (12 mg).

### 3.4. Glochidpurnoid A (***1***)

White powder; ${\left[\alpha \right]}_{D}^{25}$ + 38.0 (*c* 0.1, MeCN); UV (MeCN) *λ*_max_ (log *ε*) 309 (2.24), 295 (2.23) nm; ECD (*c* 1.5 × 10^−4^ M, MeCN) *λ*_max_ (Δ*ε*) 203 (+ 3.01) nm; IR (KBr) *ν*_max_ 2953, 2924, 2853, 1690, 1605, 1460, 1377, 1184, 1166, 828, 524 cm^−1^; ^1^H and ^13^C NMR data, see Table 1; HRESIMS *m*/*z* 625.3853 [M + Na]^+^ (calcd. for C_39_H_54_O_5_Na^+^, 625.3863).

### 3.5. Glochidpurnoid B (**2**)

White powder; ${\left[\alpha \right]}_{D}^{25}$ + 24.5 (*c* 0.1, MeCN); UV (MeCN) *λ*_max_ (log *ε*) 321 (4.28), 294 (3.75) nm; ECD (*c* 2.1 × 10^−4^ M, MeCN) *λ*_max_ (Δ*ε*) 203 (+ 6.92) nm; IR (KBr) *ν*_max_ 2950, 2925, 2866, 1694, 1632, 1513, 1453, 1376, 1265, 1169, 736, 571 cm^−1^; ^1^H and ^13^C NMR data, see Table 1; HRESIMS *m*/*z* 655.3972 [M + Na]^+^ (calcd. for C_40_H_56_O_6_Na^+^, 655.3969).

### 3.6. X-ray Crystal Structure Analysis of ***3***

Compound **3** was recrystallized from methanol to afford colorless needles. X-ray data were collected using an Angilent Xcalibur Nova X-ray diffractometer and analyzed using olex2. C_34_H_58_O_6_, *M* = 562.80 g/mol, orthorhombic, space group P2_1_2_1_2_1_ (no. 19), *α* = 7.72977(5) Å, *b* = 15.66393(13) Å, *c* = 26.4984(2) Å, *V* = 3208.339(4) Å^3^, *Z* = 4, *T* = 100.00(10) K, *μ* (Cu K*α*) = 0.613 mm^−1^, *D_calc_* = 1.165 g/cm^3^, 33,138 reflections measured (6.556° ≤ 2θ ≤ 157.354°), 6797 unique (*R*_int_ = 0.0454, *R*_sigma_ = 0.0340) which were used in all calculations. The final *R*_1_ was 0.0374 (I > 2*σ*(I)) and *wR*_2_ was 0.0974 (all data). Flack parameter = −0.01 (6). Crystallographic data for the structure of **3** have been included in the Cambridge Crystallographic Data Center (CCDC number: 2215966).

### 3.7. Alkaline Hydrolysis of ***1**–**3***

Potassium carbonate drying granules (5 mg) and a drop of water were added into a stirred solution of **3** (5 mg) in CH_3_OH (2 mL). After stirring at rt for 6 h, the organic phase was extracted and evaporated in vacuo to obtain the hydrolysis product **3a** (2.0 mg). Correspondingly, sodium ethoxide drying granules (5 mg) and a drop of water were added into a solution of **2** (5 mg) in CH_3_OH (2 mL). The reaction was stirred at 120 °C for 2 h and then directly subjected to HPLC (CH_3_CN/H_2_O, 90%, 3 mL/min) to obtain hydrolysis product **2a** (1.6 mg). Treating **1** in the same way as **2** afford the hydrolysis product **1a** (1.3 mg). These hydrolysis products **1a**–**3a** were finally identified as the same compound, morolic acid [19], by comparison of their ^1^H NMR data.

### 3.8. Cell Culture

The HCT-116 (human colorectal carcinoma cell line) cells were purchased from the Laboratory Animal Service Center at Sun Yat-sen University (Guangzhou, China). Cells were maintained in 1640 medium containing 10% FBS (Gibco BRL, Waltham, MA, USA) and incubated at 37 °C in a cell incubator with 5% CO_2_.

### 3.9. Cell Viability Assay

MTT assay was applied to examine cytotoxicity of the candidates in HCT-116 cells. Cells seeded into 96-well plates at a density of 1 × 10^3^ cells/well were incubated for 24 h, then cells were treated with or without indicated concentrations of the candidates for 48 or 96 h. After incubation, 10 μL per well of MTT solution (5 mg/mL) was supplied and incubated for 4 h. After discarding the suspension, each well was added into 100 μL of DMSO, and the absorbance was determined by a multifunction microplate reader (Biotek., USA) at 570 nm. The assays were carried out in triplicate.

### 3.10. Colony Formation Assay

HCT-116 cells were plated in 6-well plates at a density of 1 × 10^3^ cells/well for 24 h and later added into various concentrations of **2**. Cells incubated for 12 days were fixed with 4% paraformaldehyde (Biosharp, Hefei, China) for 30 min and stained with Ciemsa stain (Beyotime, Haimen, China).

### 3.11. Western Blot Analysis

The cells were seeded evenly at a density of 2 × 10^5^ cells/well in 6-well plates and were allowed to attach for 24 h. After treated with **2** for 12 h, the cells were rinsed three times with ice-cold PBS and lysed in RIPA buffer (Beyotime, China) containing protease inhibitor cocktails (Roche Life Science, Branford, CT, USA). The lysates were centrifugated at 12,000× *g* for 30 min at 4 °C, and the total protein concentration was determined using a BCA protein assay kit (Beyotime, China). The cell lysates were mixed with sample dye (Cwbio, Beijing, China) and boiled at 95 °C for 10 min. The mixture was resolved by SDS-PAGE electrophoresis and transferred to PVDF membrane. The blots were blocked with TBST (Tris-Buffered Saline Tween-20) containing 5% skim milk for 2 h. The antibodies were diluted using a Primary Antibody Dilution Buffer (Beyotime, China) at a ratio of 1:1000, and the blots were incubated with specific antibodies at 4 °C for 12 h. Subsequently, the blots were detected using enhanced chemiluminescence detection kit (Thermo, Waltham, MA, USA). The density of the immunoblot was analyzed using ImageJ software (National Institute of Health, Bethesda, MD, USA).

## 4. Conclusions

Generally, diterpenoids are recognized as the characteristic metabolites of the Euphorbiaceae plants [37]. Interestingly, previous and current chemical investigation suggested that triterpenoids were the main metabolites of *G. puberum*. In the current study, two new oleanolanes, designated as glochidpurnoid A (**1**) and B (**2**), along with seventeen known lupane triterpenoids were isolated from the whole herbs of G. *purberum*, of which six compounds showed high cytotoxicity against HCT-116 cells. Among them, **2**, a new oleanane possessing a feruloyl moiety at OH-3, showed the most pronounced activity with IC_50_ = 0.80 ± 0.05 μM, being more potent than the positive control 5-FU. Compared with **1** and **3**, it seems that the *trans*-feruloyl substituent group in **2** is a key pharmacophore for the activity. Mechanistic study implied that **2** could induce apoptosis in HCT-116 cells via ER stress signaling and improve the sensitivity of CRC to 5-FU. Notably, although limited amount of **2** was found from the nature, the easily obtained *trans*-ferulic acid and morolic acid may provide an opportunity for us to mass-produce **2** by semi-synthesis and verify its in vivo anti-CRC effect in the future. This research not only enlarges the structural diversity of triterpenoids from the Euphorbiaceae, but also provides a potent agent for further developing anti-CRC drugs.

## Figures and Tables

**Table 1:** ^1^H (400 MHz) and ^13^C (100 MHz) NMR data for **1** and **2** (*δ* in ppm).

No.	1 ^a^		2 ^b^	
	*δ*_H_, Multi. (*J* in Hz)	*δ*_C_, Type	*δ*_H_, Multi. (*J* in Hz)	*δ*_C_, Type
1	*α* 0.92, m *β* 1.65, m	39.0, CH_2_	*α* 1.06, m *β* 1.75, m	38.6, CH_2_
2	*α* 1.88, m *β* 1.79, m	24.6, CH_2_	a 1.73, m b 1.73, m	23.8, CH_2_
3	4.90, dd (11.6, 4.7)	80.9, CH	4.63, dd (10.5, 5.5)	80.8, CH
4		38.6, C		38.1, C
5	0.87, m	56.1, CH	0.85, d (9.7)	55.6, CH
6	*α* 1.31, m *β* 1.49, m	18.8, CH_2_	*α* 1.51, m *β* 1.37, m	18.1, CH_2_
7	a 1.43, m b 1.34, m	35.2, CH_2_	a 1.47, m b 1.35, m	34.5, CH_2_
8		41.3, C		40.7, C
9	1.34, m	51.7, CH	1.30, m	51.1, CH
10		37.7, C		37.1, C
11	a 1.49, m b 1.21, m	21.7, CH_2_	*α* 1.57, m *β* 1.28, m	20.9, CH_2_
12	*α* 1.33, m *β* 1.73, m	26.8, CH_2_	a 1.62, m b 1.25, m	26.0, CH_2_
13	2.72, d (11.2)	42.0, CH	2.22, d (9.7)	41.3, CH
14		43.4, C		42.5, C
15	*α* 1.46, m *β* 2.03, m	30.3, CH_2_	a 1.68, m b 1.23, m	29.4, CH_2_
16	*α* 1.33, m *β* 1.82, m	34.7, CH_2_	a 2.16, m b 1.40, m	33.5, CH_2_
17		48.9, C		47.9, C
18		139.3, C		136.7, C
19	5.33, s	132.5, CH	5.17, s	133.2, CH
20		32.8, C		32.0, C
21	a 1.46, m b 1.46, m	34.6, CH_2_	a 1.99, m b 1.40, m	33.4, CH_2_
22	a 2.36, m b 2.36, m	34.6, CH_2_	a 2.01, m b 1.65, m	33.3, CH_2_
23	0.97, s	28.5, CH_3_	0.89, s	28.0, CH_3_
24	1.04, s	17.3, CH_3_	0.92, s	16.7, CH_3_
25	0.82, s	17.1, CH_3_	1.00, s	16.0, CH_3_
26	0.99, s	16.6, CH_3_	0.91, s	16.7, CH_3_
27	0.99, s	15.7, CH_3_	0.79, s	14.9, CH_3_
28		179.4, C		182.2, C
29	1.16, s	31.2, CH_3_	1.00, s	30.3, CH_3_
30	1.10, s	29.7, CH_3_	0.98, s	29.1, CH_3_
1′		167.7, C		167.2, C
2′	6.74, d (15.9)	116.2, CH	6.29, d (15.9)	116.2, CH
3′	8.07, d (15.9)	145.4, CH	7.59, d (15.9)	144.4, CH
4′		126.6, C		127.1, C
5′	7.69, d (8.6)	131.1, CH	7.03, d (1.7)	109.3, CH
6′	7.20, m	117.3, CH		146.7, C
7′		161.8, C		147.8, C
8′	7.20, m	117.3, CH	6.91, d (8.2)	114.7, CH
9′	7.69, d (8.6)	131.1, CH	7.07, dd (8.2, 1.7)	123.0, CH
6′-OCH_3_			3.93, s	55.9, CH_3_

^a^ Measured in pyridine-*d*_5_; ^b^ Measured in CDCl_3_.

**Table 2:** Cytotoxic effects of **1**–**19** against HCT-116 cells ^a^.

Compound	IC_50_ (μM)		Compound	IC_50_ (μM)	
	48 h	96 h		48 h	96 h
**2**	1.90 ± 0.02	0.80 ± 0.05	**11**	5.30 ± 0.59	2.91 ± 0.06
**3**	7.12 ± 0.60	2.99 ± 0.34	**17**	5.33 ± 0.18	2.40 ± 0.47
**5**	2.75 ± 0.05	1.89 ± 0.02	5-FU ^b^	13.50 ± 0.59	3.79 ± 0.17
**6**	2.40 ± 0.71	1.16 ± 0.07			

^a^ Compounds with IC_50_ > 50 μM were not listed. ^b^ Positive control.

## Data Availability

All relevant data have been provided within the manuscript.

## Supplementary Materials

The following supporting information can be downloaded at: https://www.mdpi.com/article/10.3390/molecules28020511/s1, Content S1: CD, IR, and HRESIMS spectra of **1** and **2**. Content S2: 1D and 2D NMR spectra of **1** and **2**. Supplementary method S3: Details of chemical procedures and instruments.

## Sample Availability

Samples of the compounds are available from the authors upon request.
