# Supplementary material for: Glochodpurnoid B from Glochidion puberum Induces Endoplasmic Reticulum Stress-Mediated Apoptosis in Colorectal Cancer Cells
Source: Molecules. 2023 Jan 4;28(2):511. doi: 10.3390/molecules28020511 (PMC9867043; doi:10.3390/molecules28020511)
Supplement: Supplementary file 1 [file molecules-28-00511-s001.zip › Supporting Information-Molecules.pdf]

Glochodpurnoid B from *Glochidion puberum* induces endoplasmic reticulum stress-mediated apoptosis in colorectal cancer cells

Yang Tian [1†], Runzhu Fan [1†], Zhao Yin [2], Yongping Huang [3], Dong Huang [1], Fangyu Yuan [1], Aiping Yin [4], Guihua Tang [1], Rong Pu [4*], Sheng Yin [1*]

[1] *School of Pharmaceutical Sciences, Sun Yat-sen University, Guangzhou, Guangdong 510006, China*

[2] *Department of Geratology, The Affiliated Hospital of Yunnan University, The Second People's Hospital of Yunnan Province, Kunming, Yunnan 650021, China*

[3] *Hanshan Normal University, Chaozhou, Guangdong 521041, China*

[4] *Department of Clinical Laboratory, The Third People's Hospital of Dongguan, Dongguan 523326, China*

[†]These authors have contributed equally to this work.

*Corresponding authors

*(S. Yin) Tel: +86-20-39943090. E-mail: yinsh2@mail.sysu.edu.cn.

*(R. Pu) Tel: +18002900838. E-mail: purong328073@163.com.

Contents:

S1. CD, IR, and HRESIMS spectra of **1** and **2**……………………….........……P3-P5

**S1.1.** CD data of compound **1**	**S1.2.** HRESIMS data of compound **1**
**S1.3.** IR data of compound **1**	**S1.4.** CD data of compound **2**
**S1.5.** HRESIMS data of compound **2**	**S1.6.** IR data of compound **2**

S2. 1D and 2D NMR spectra of **1** and **2**…….............…………………........P6-P17

**S2.1.** ^{1}H NMR spectrum of compound **1**	**S2.2.** ^{13}C and DEPT 135 NMR spectra of **1**
**S2.3.** HSQC spectrum of compound **1**	**S2.4.** HMBC spectrum of compound **1**
**S2.5.** ^{1}H–^{1}H COSY spectrum of compound **1**	**S2.6.** NOESY spectrum of compound **1**
**S2.7.**^{1}H NMR spectrum of compound **2**	**S2.8.** ^{13}C and DEPT 135 NMR spectra of **2**
**S2.9.** HSQC spectrum of compound **2**	**S2.10.** HMBC spectrum of compound **2**
**S2.11.** ^{1}H–^{1}H COSY spectrum of compound **2**	**S2.12.** NOESY spectrum of compound **2**

S3. Details of chemical procedures and instruments……..............…............P18

S1.1. CD data of compound **1**

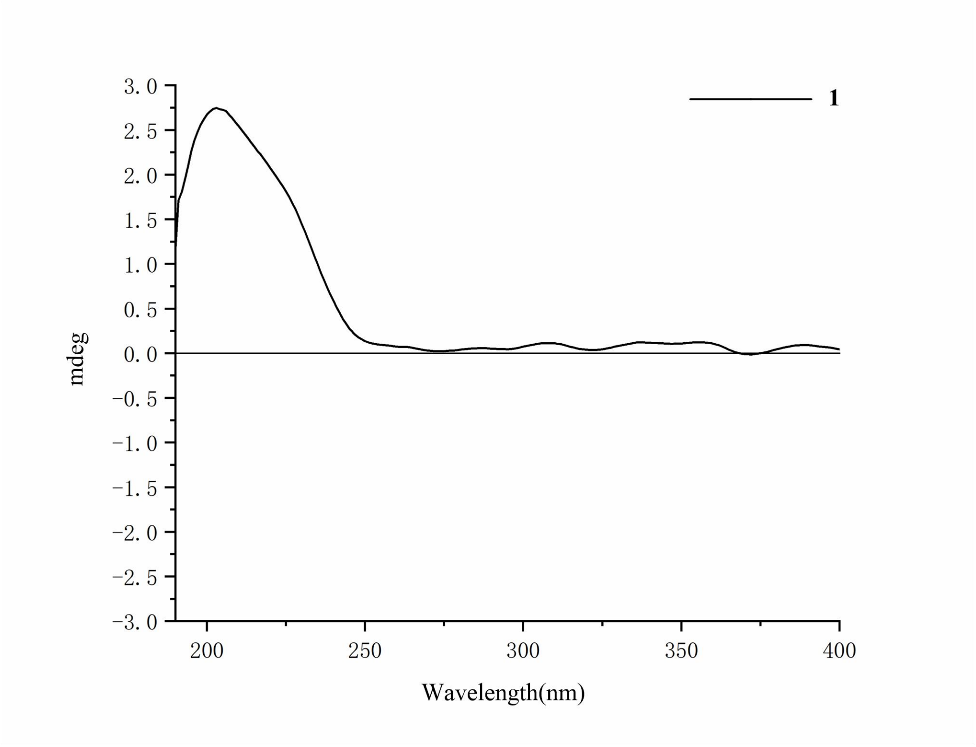

S1.2. HRESIMS data of compound **1**

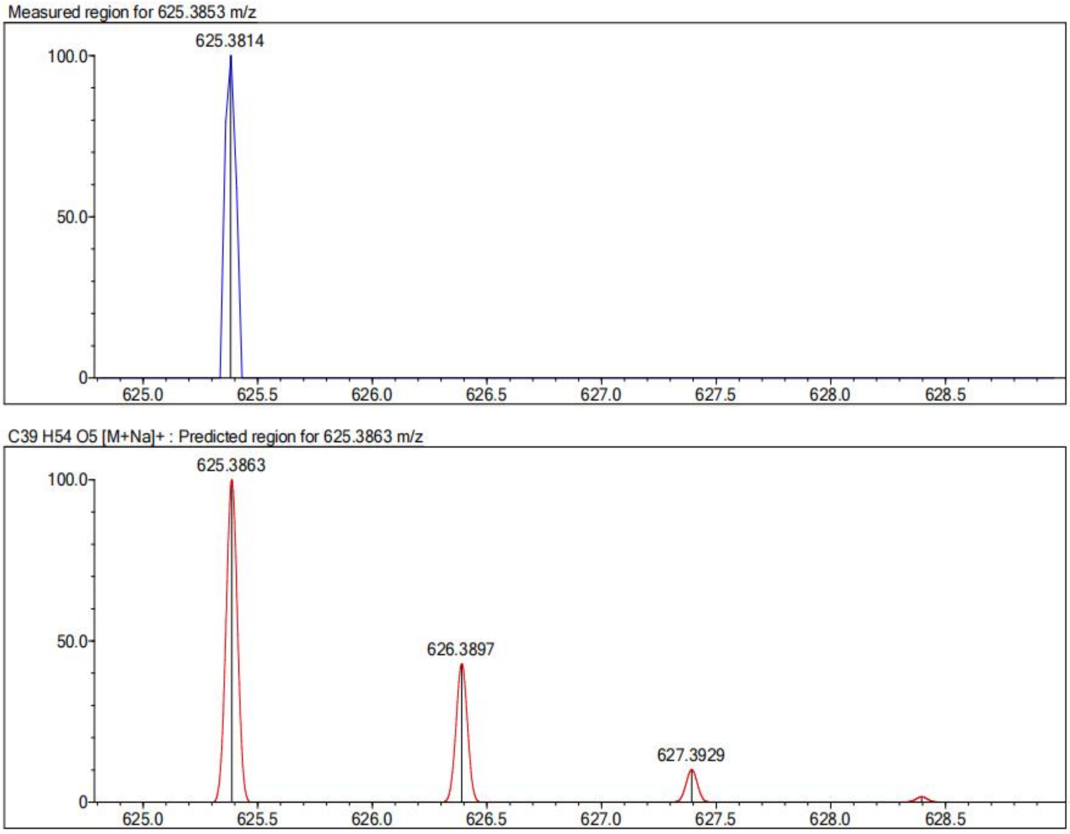

Rank	Score	Formula (M)	Ion	Meas. m/z	Pred. m/z	Df. (mDa)	Df. (ppm)	Iso	DBE
1	0.00	C39 H54 O5	[M+Na]+	625.3853	625.3863	-1.0	-1.60	0.00	13.0

S1.3. IR data of compound **1**

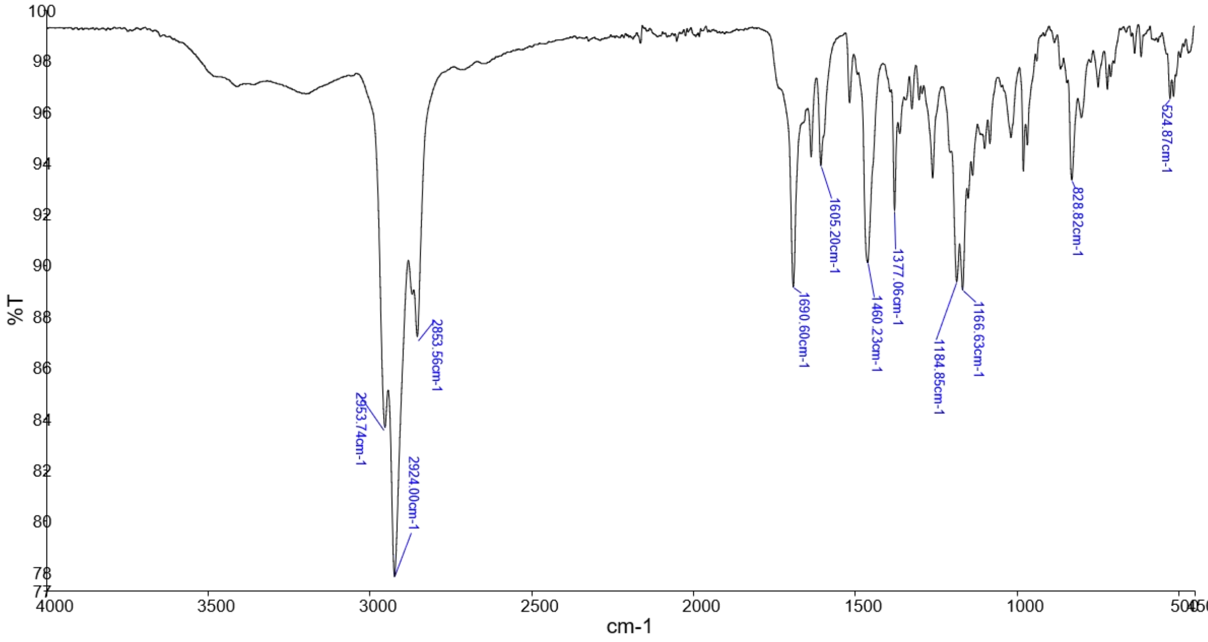

S1.4. CD data of compound **2**

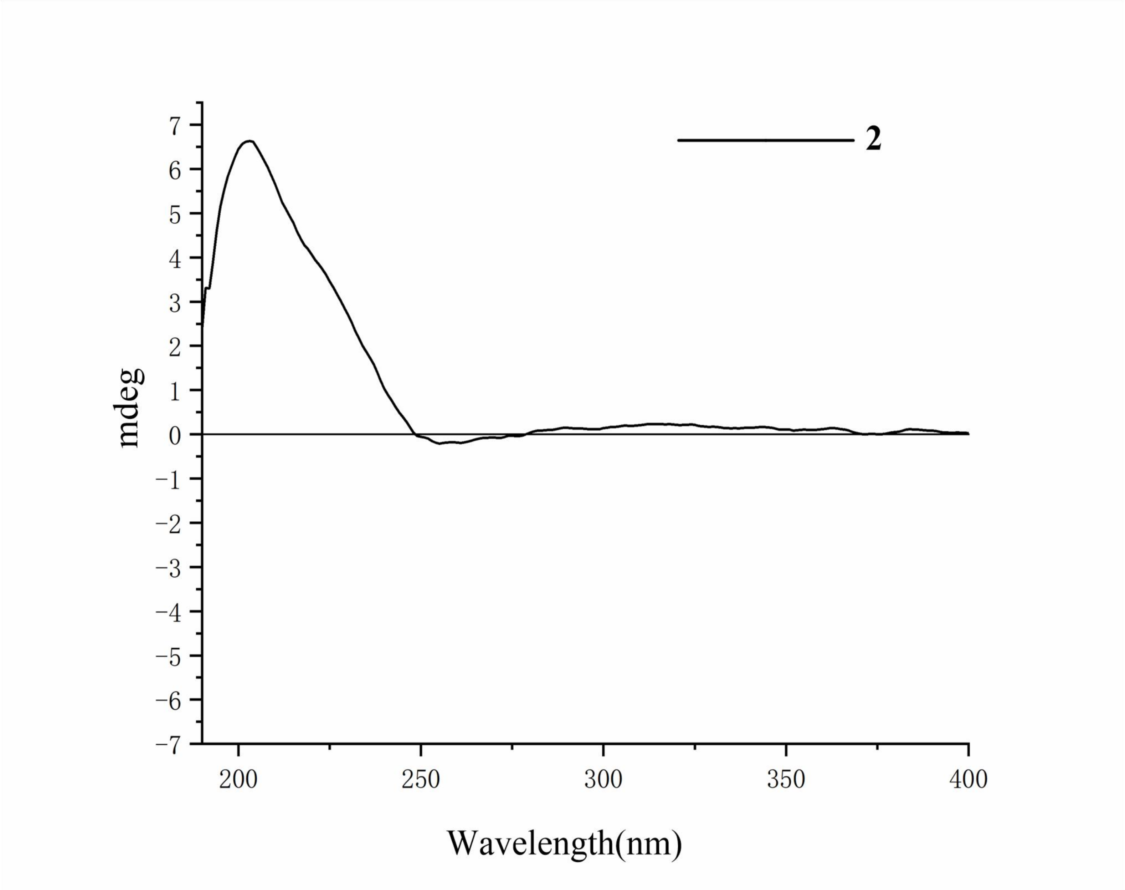

S1.5. HRESIMS data of compound **2**

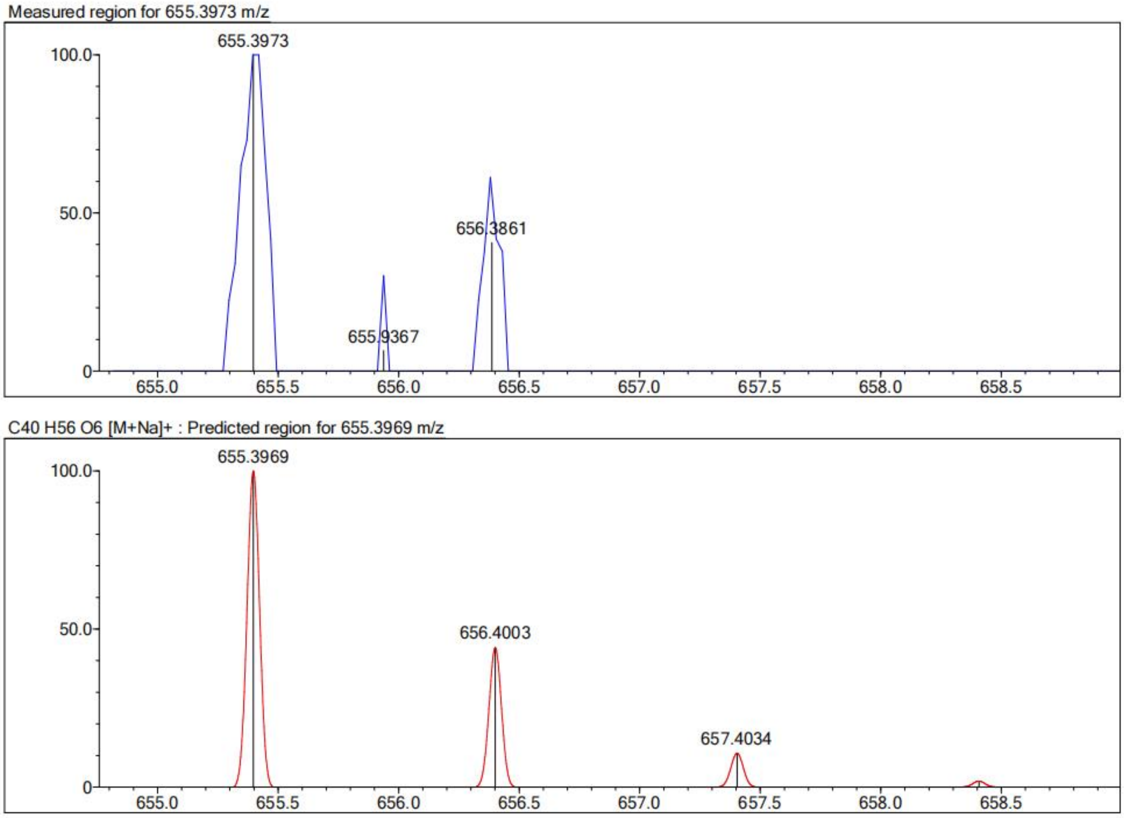

Rank	Score	Formula (M)	Ion	Meas. m/z	Pred. m/z	Df. (mDa)	Df. (ppm)	Iso	DBE
1	0.00	C40 H56 O6	[M+Na]+	655.3973	655.3969	0.4	0.61	0.00	13.0

S1.6. IR data of compound **2**

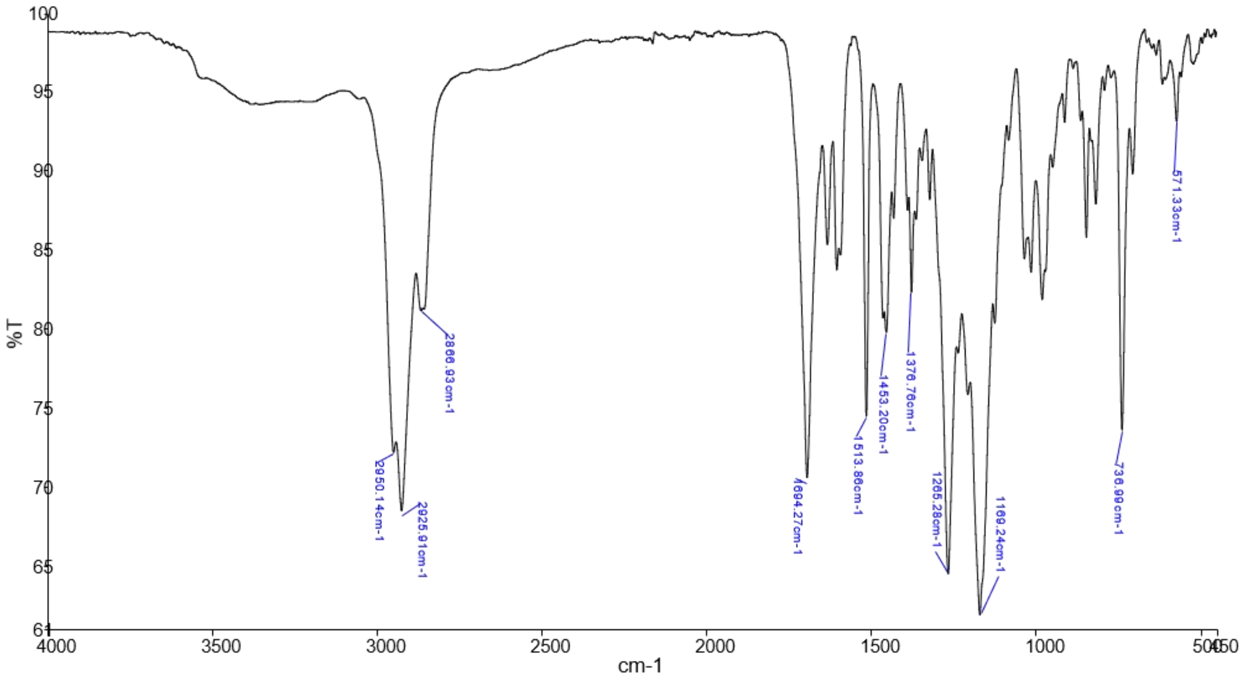

S2.1. ^{1}H NMR spectrum of compound **1**

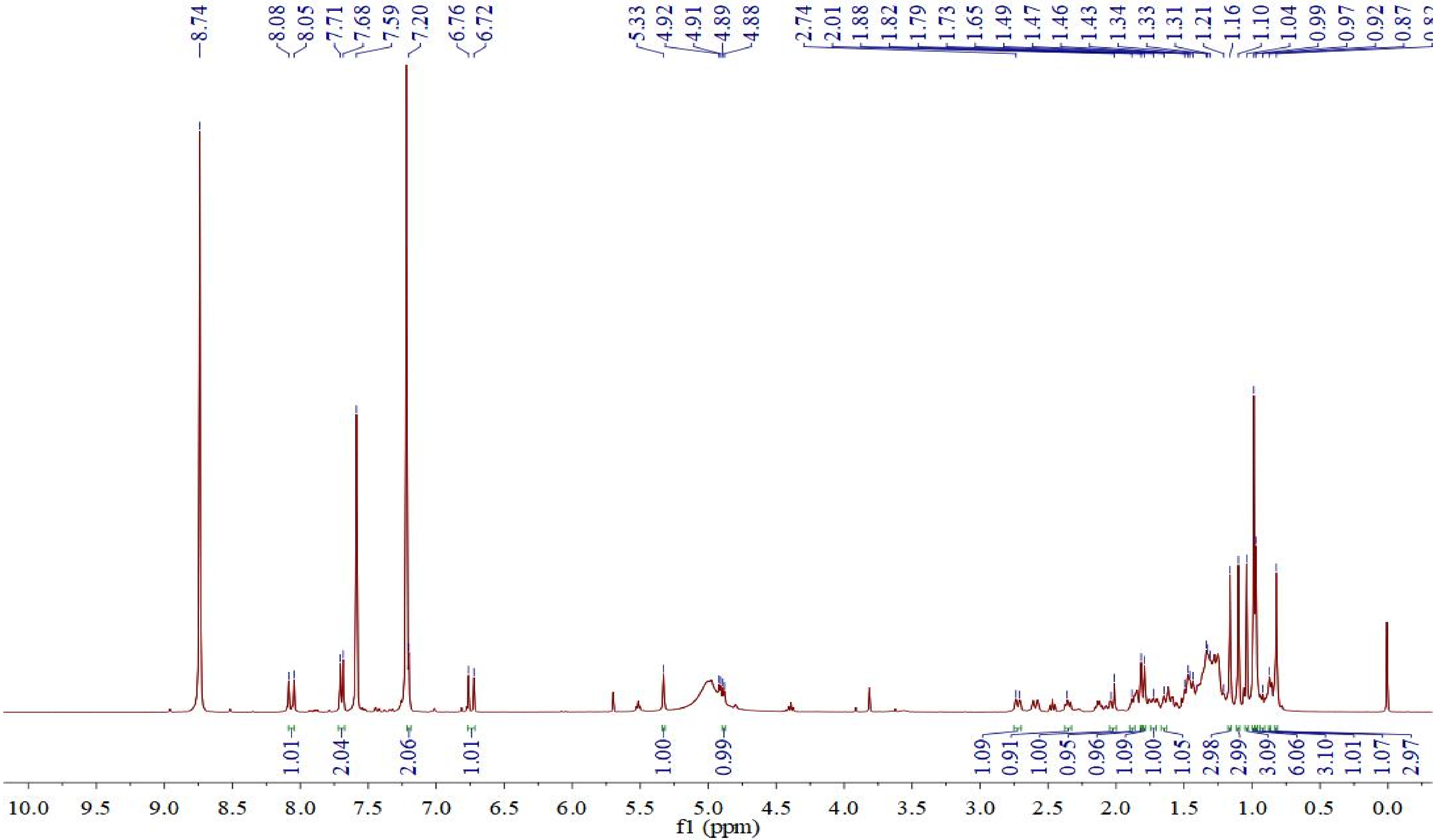

S2.2. ^{13}C and DEPT 135 NMR spectrum of compound **1**

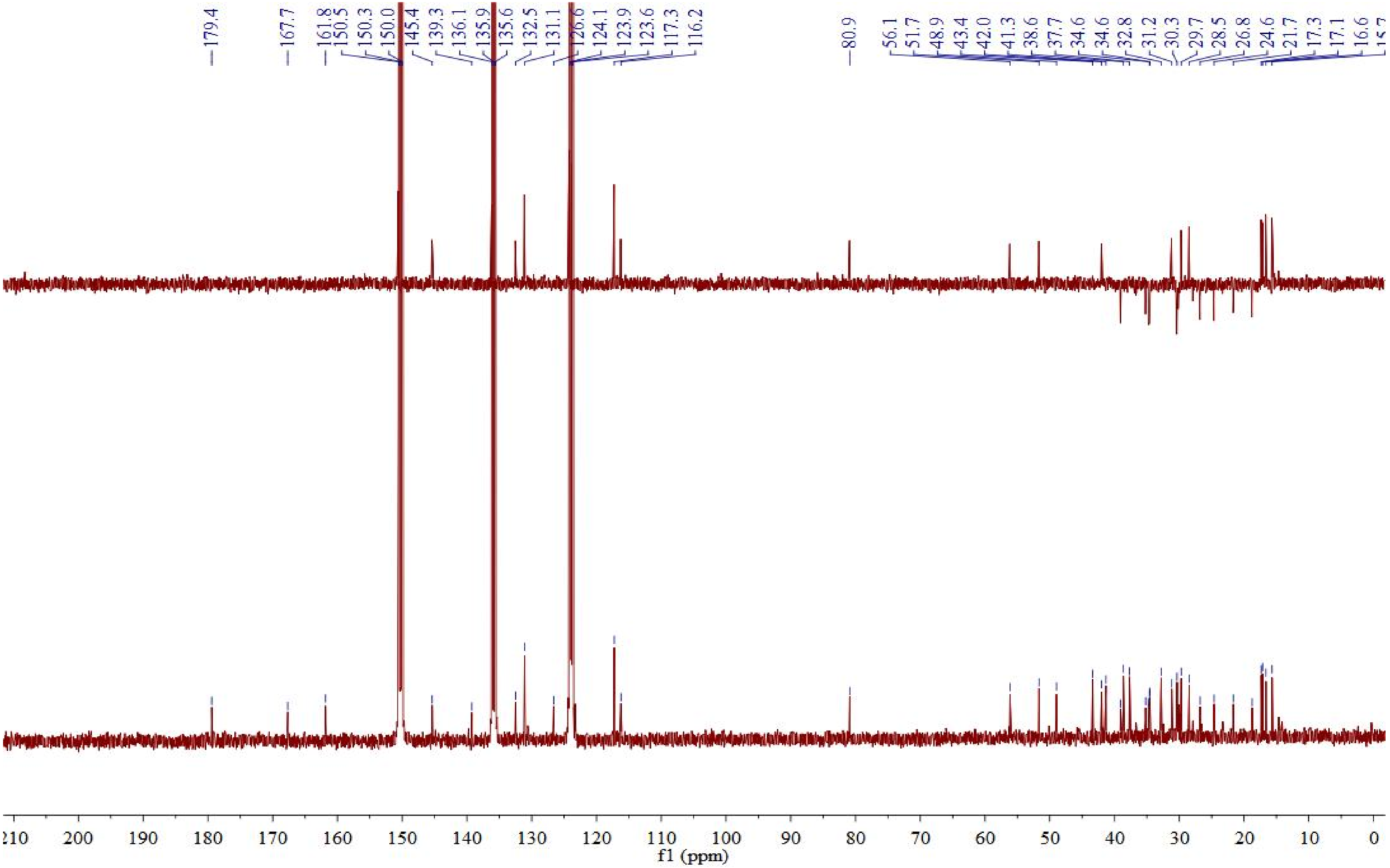

S2.3. HSQC spectrum of compound **1**

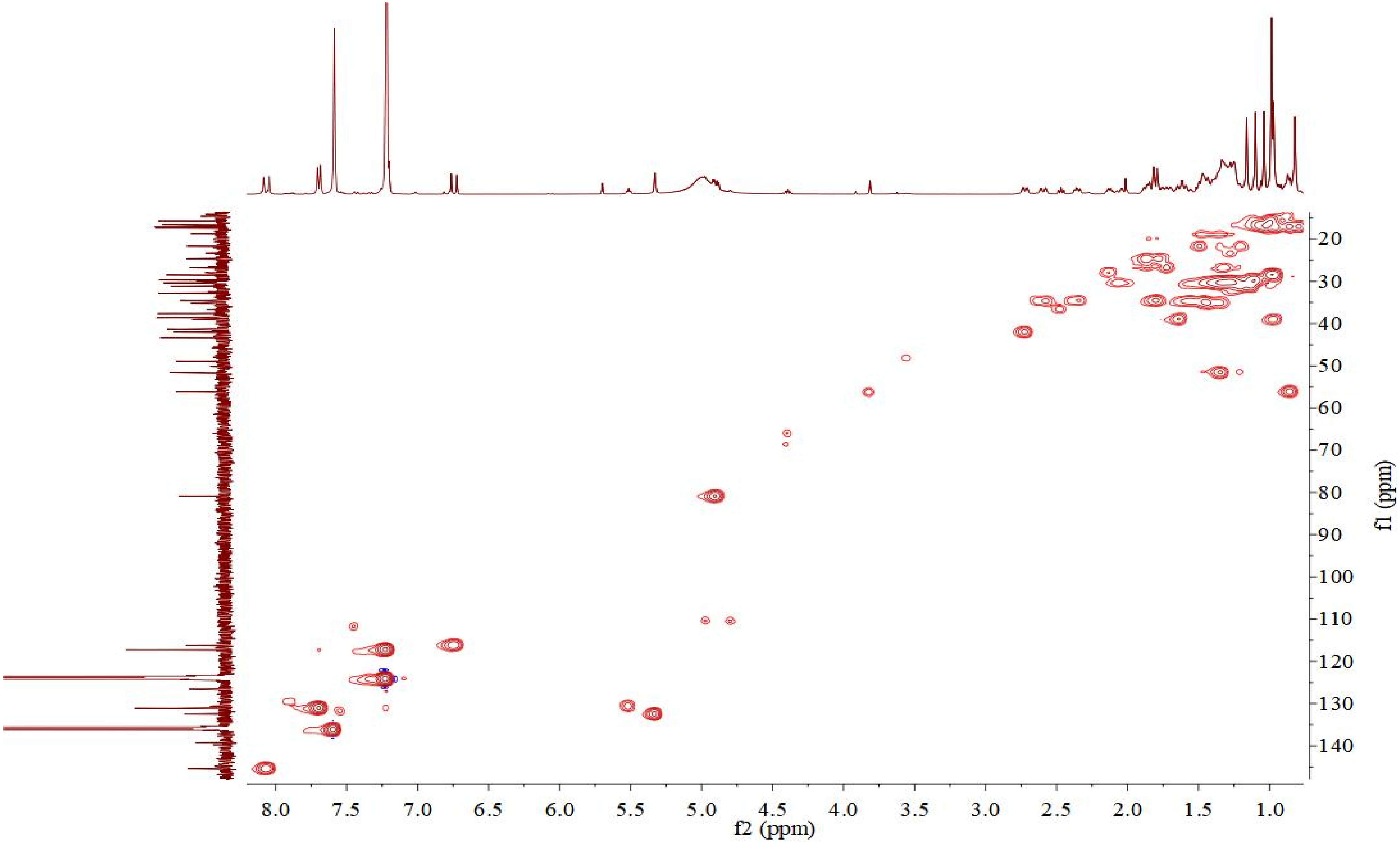

S2.4. HMBC spectrum of compound **1**

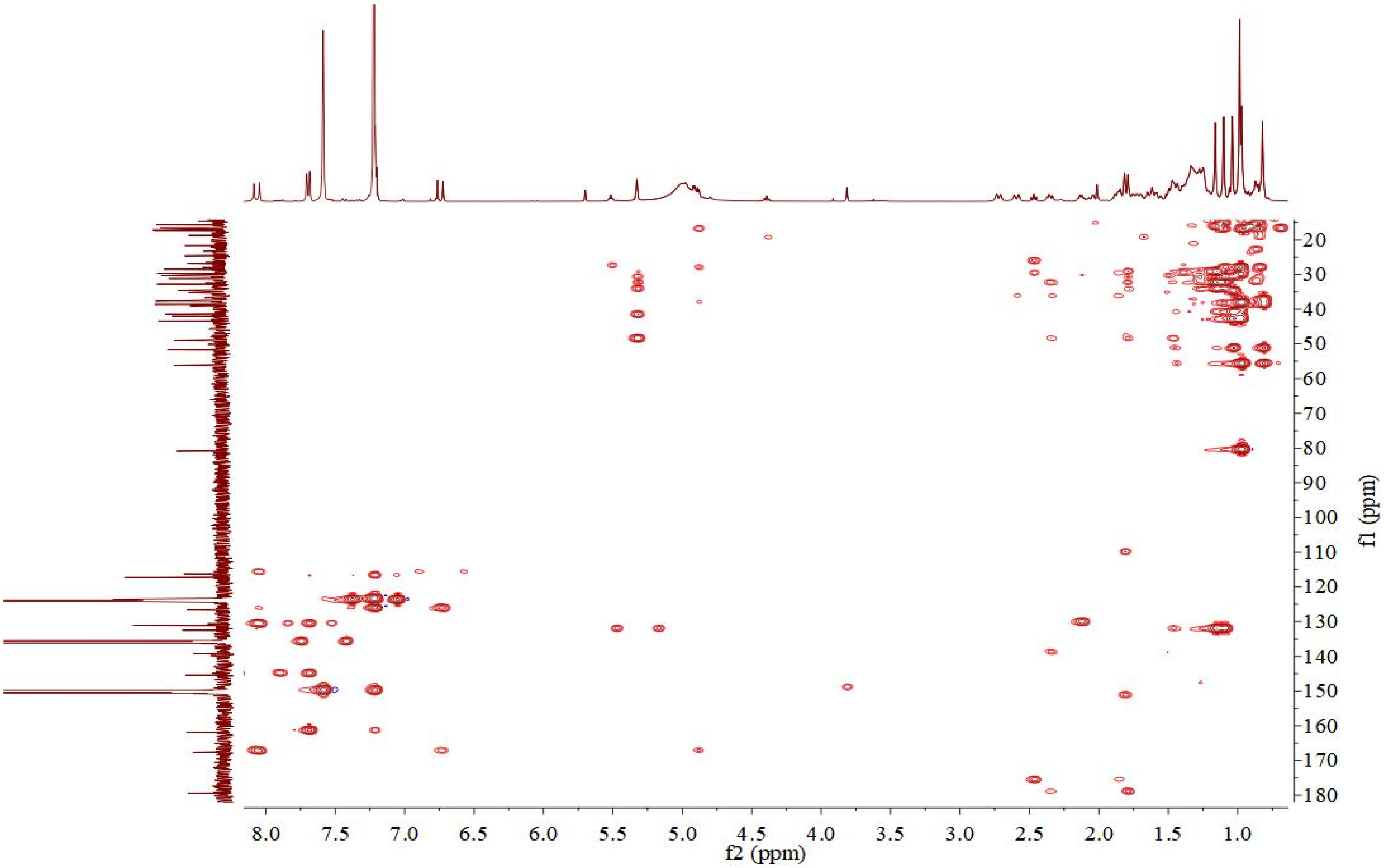

S2.5. $^1H-^1H$ COSY spectrum of compound **1**

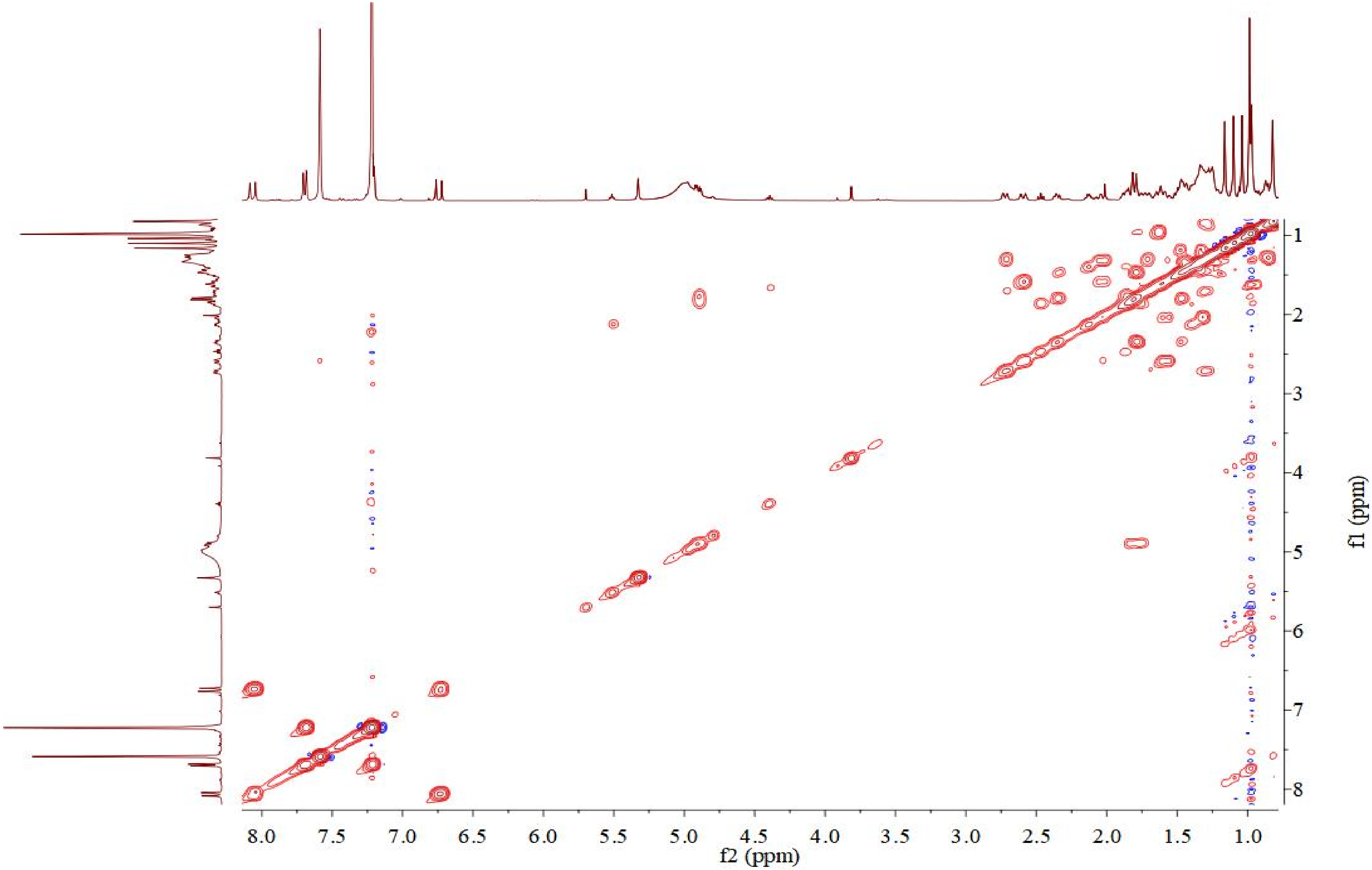

S2.6. NOESY spectrum of compound **1**

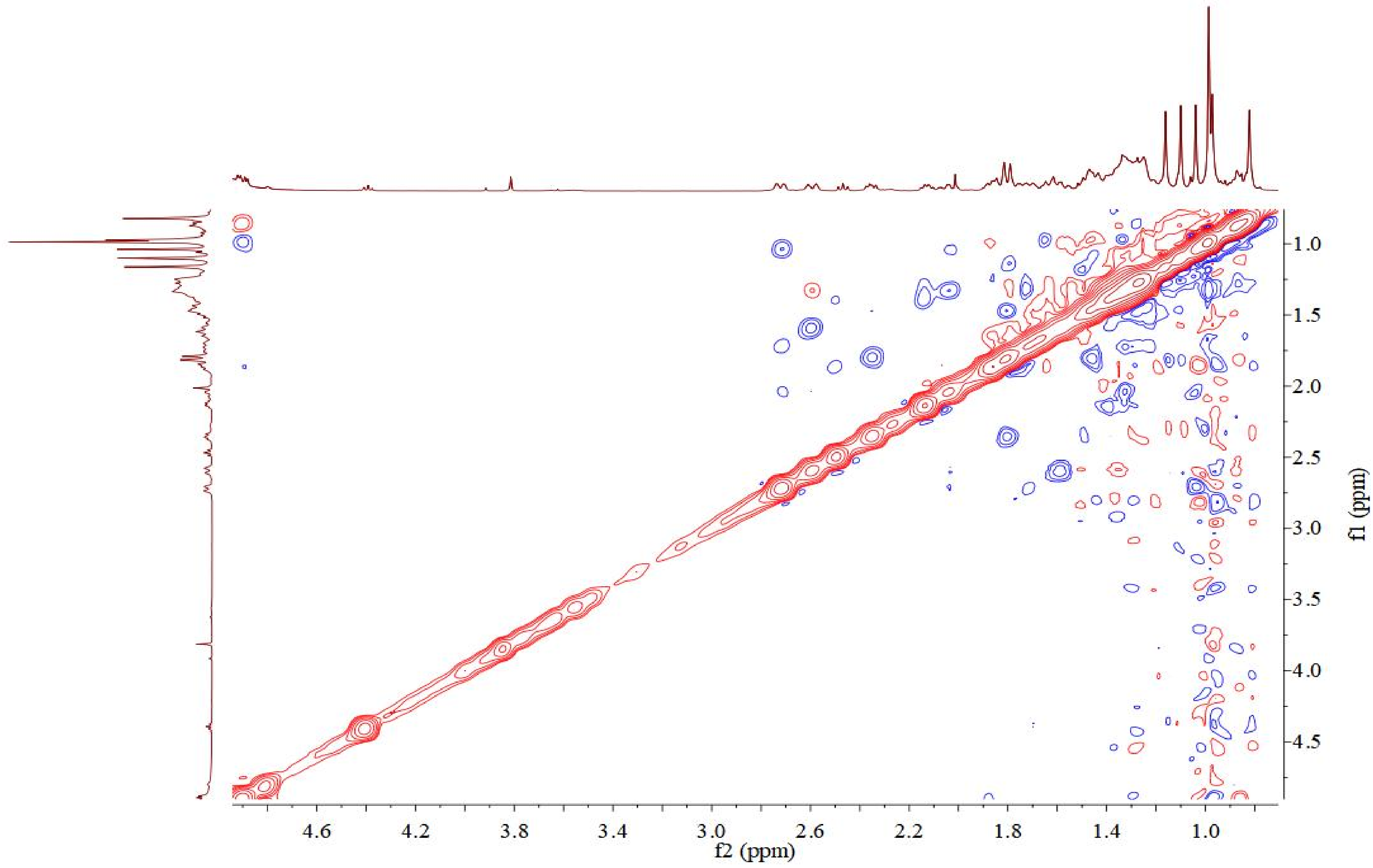

S2.7. ^{1}H NMR spectrum of compound **2**

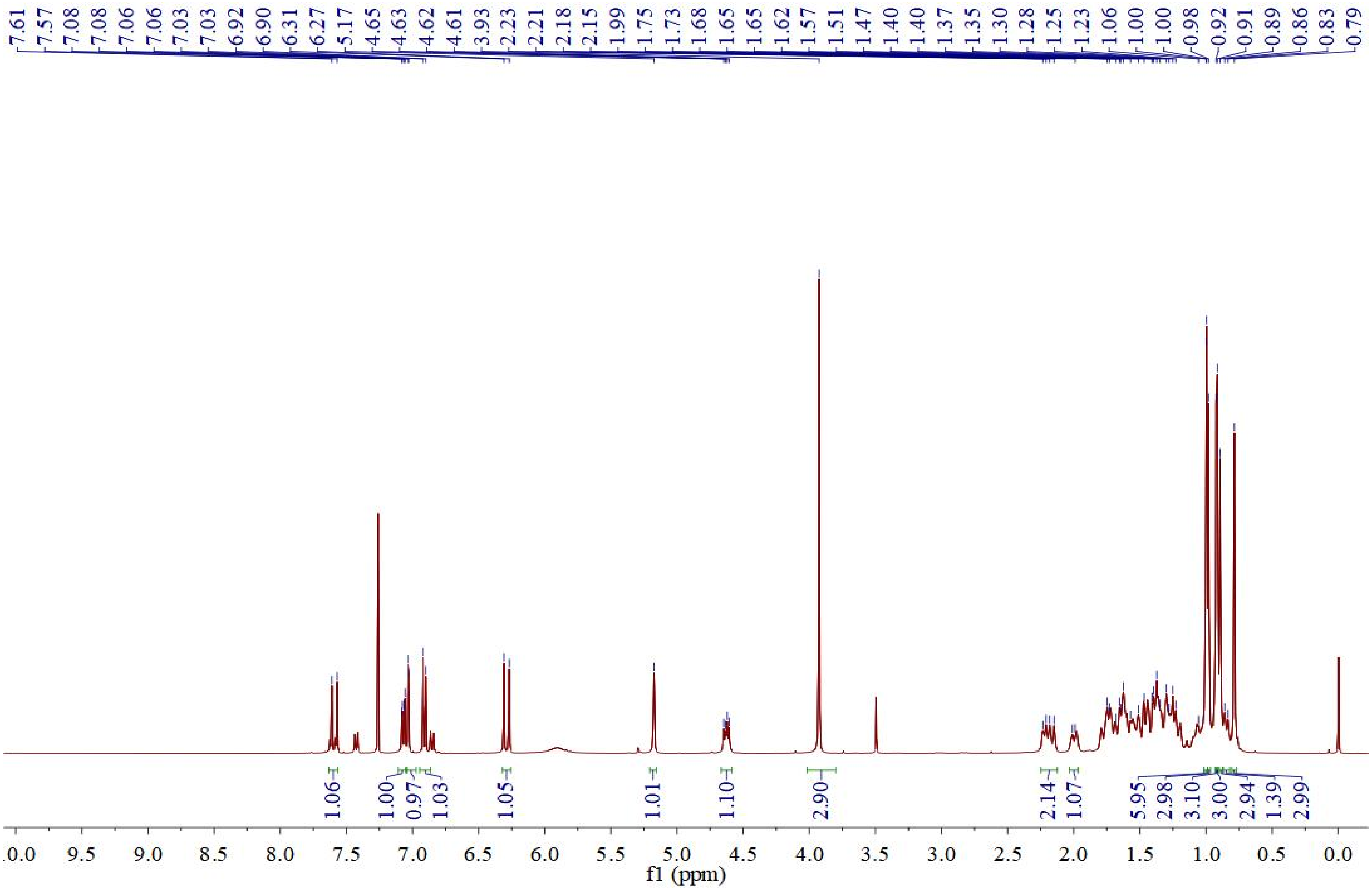

S2.8. ^{13}C and DEPT 135 NMR spectrum of compound **2**

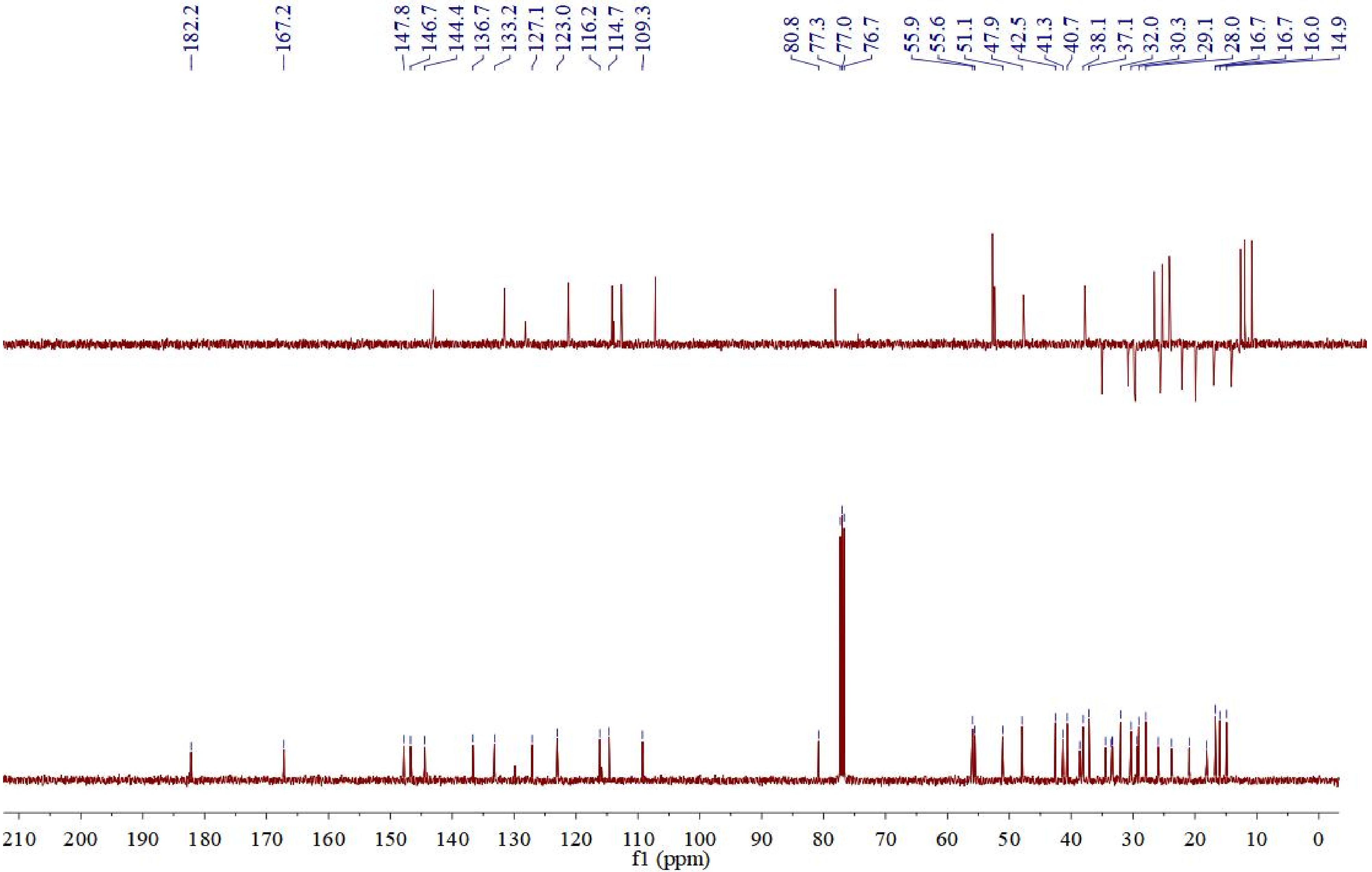

S2.9. HSQC spectrum of compound **2**

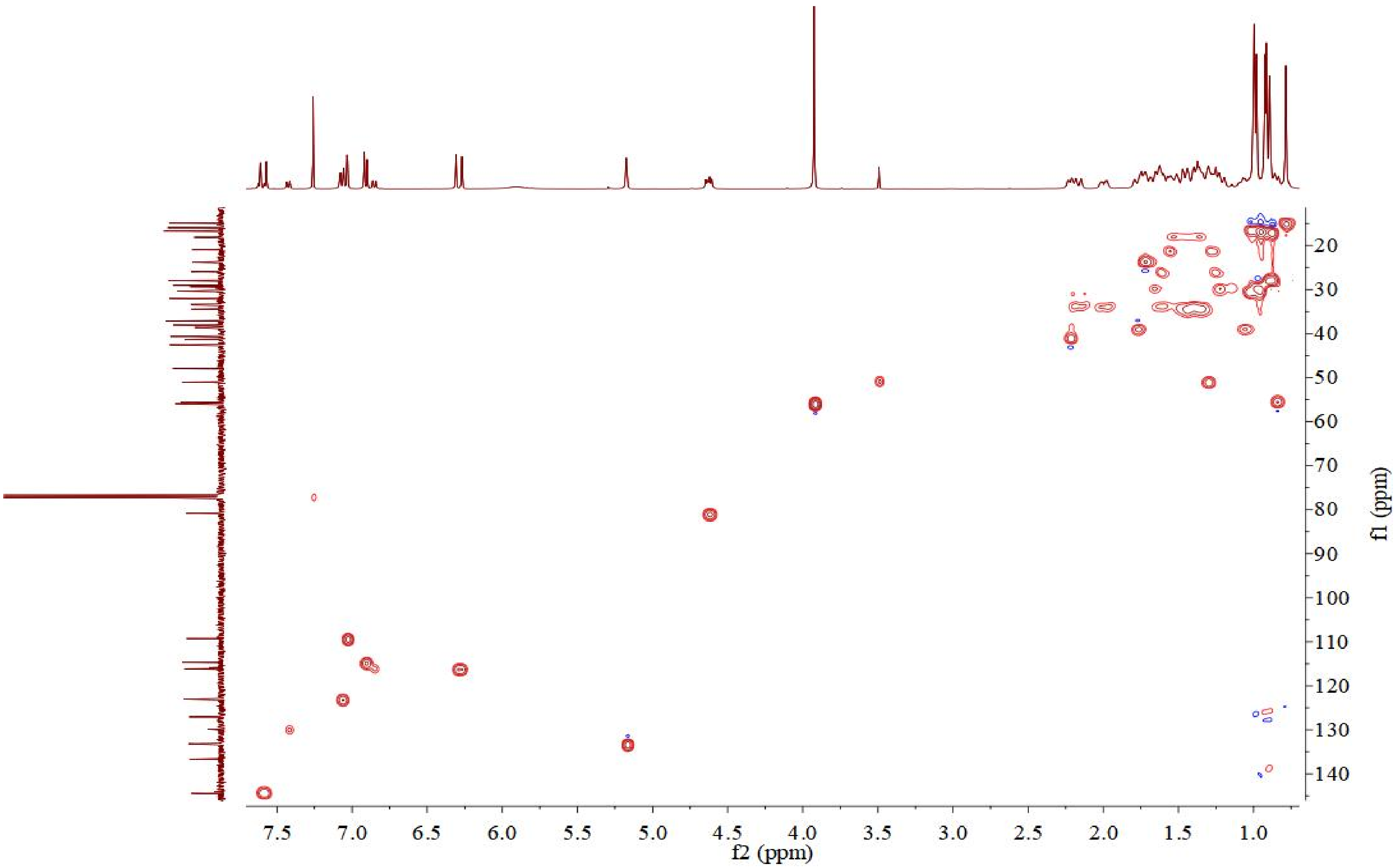

S2.10. HMBC spectrum of compound **2**

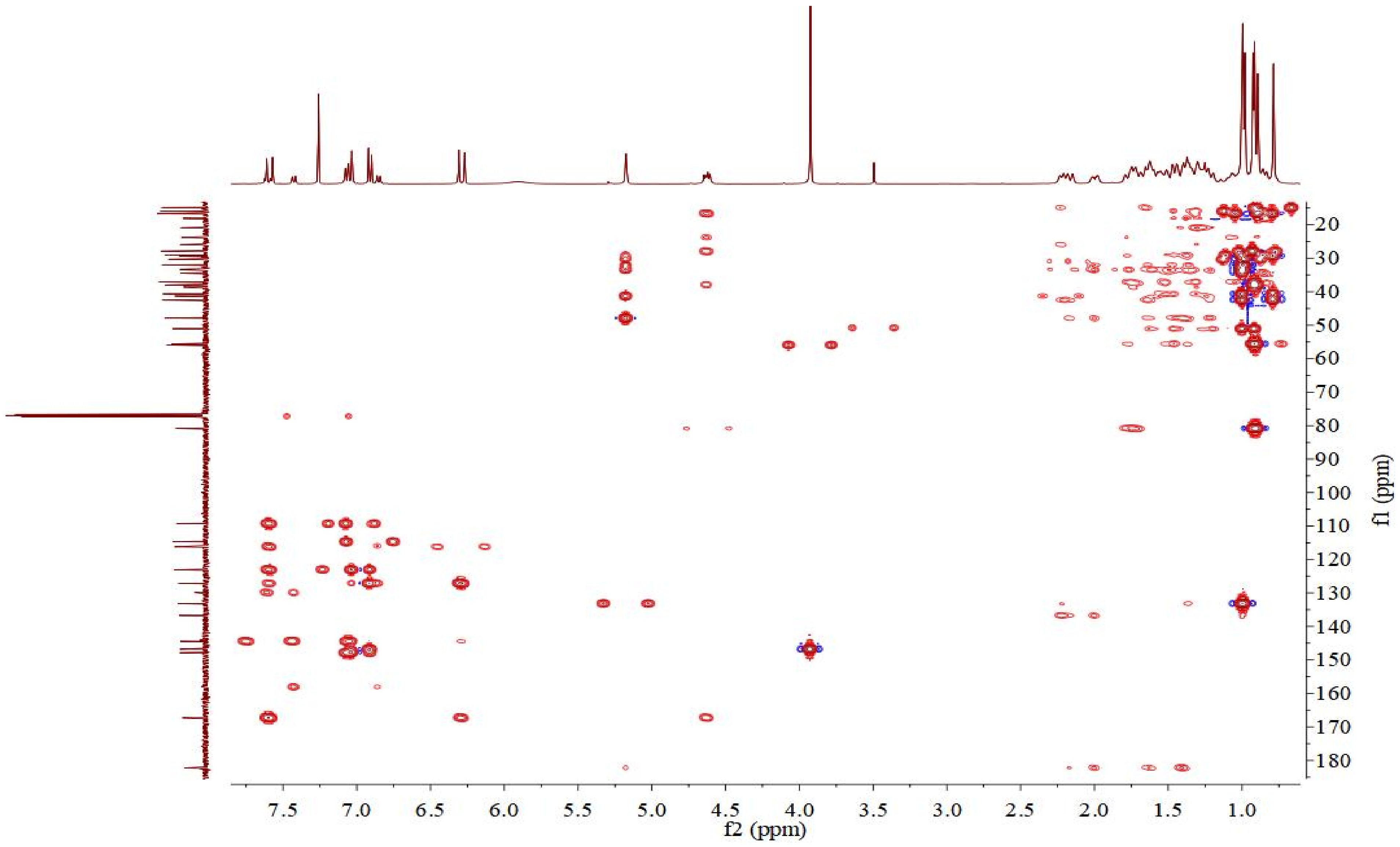

S2.11. $^{1}H-^{1}H$ COSY spectrum of compound **2**

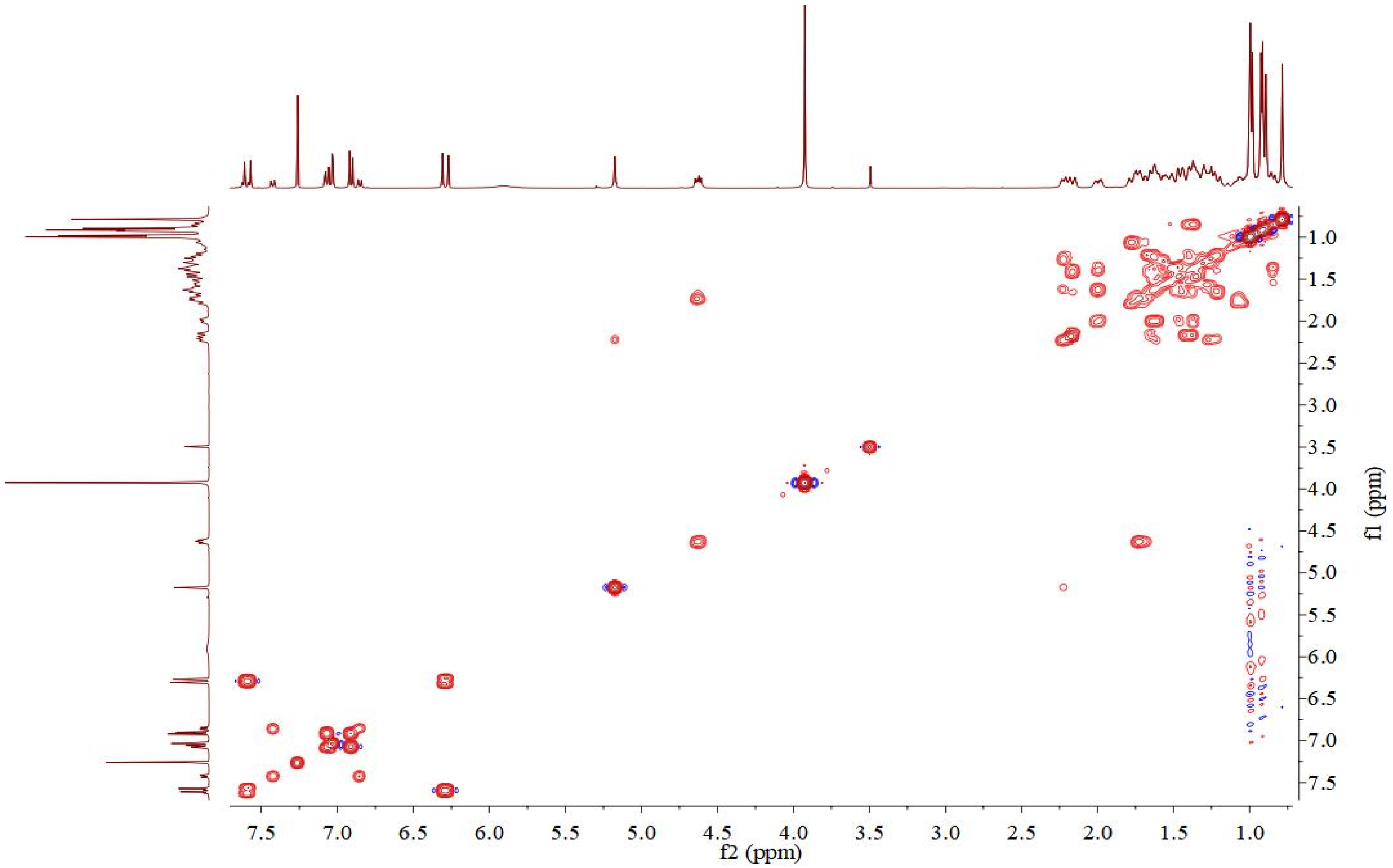

S2.12. NOESY spectrum of compound **2**

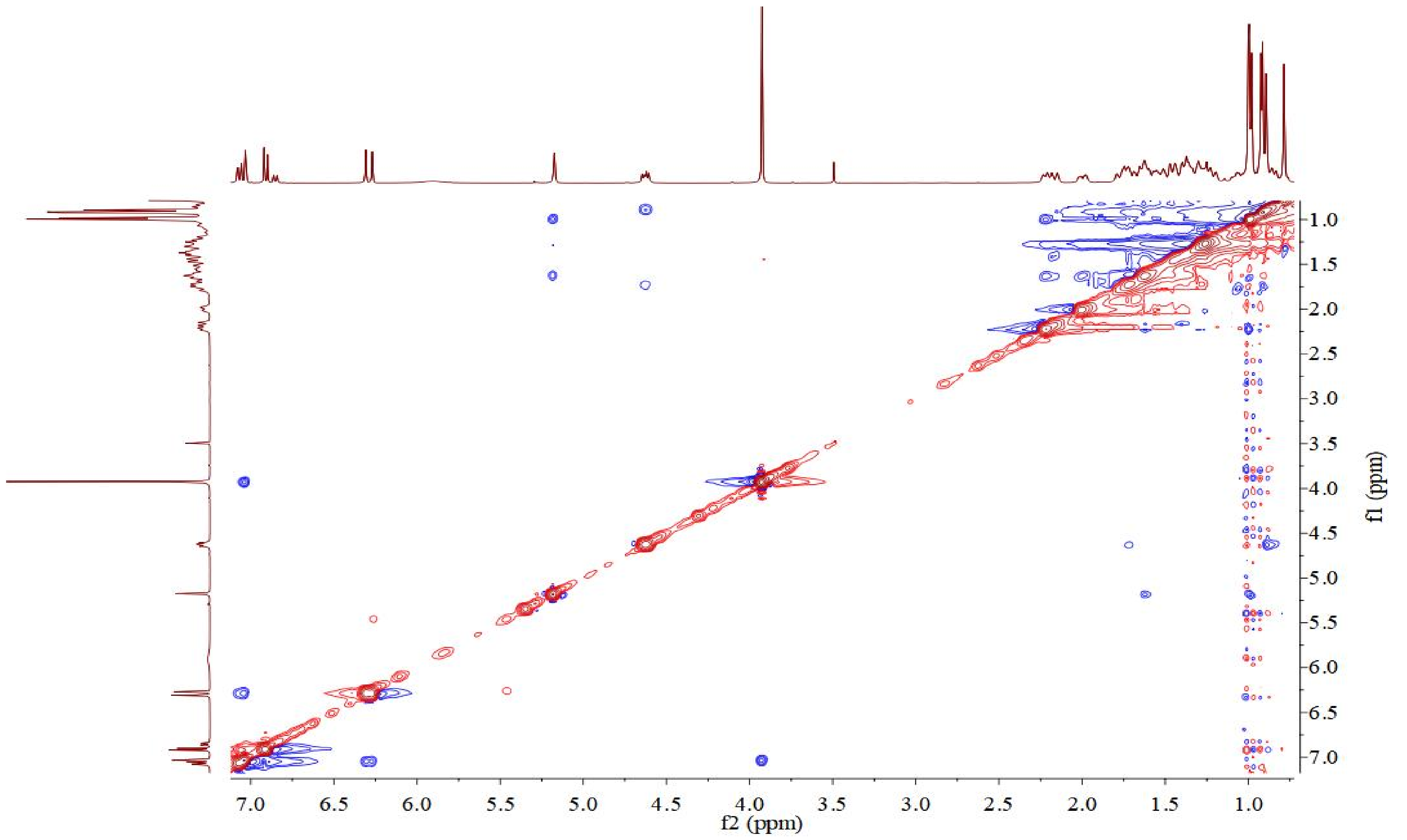

S3. Details of chemical procedures and instruments

Optical rotations were measured using a PekinElmer 341 polarimeter. HRESIMS were carried out on a Finnigan LCQ Deca instrument. ECD and UV spectra were recorded by an Applied Photophysics Chirascan spectrometer. IR spectra were measured on a Bruker Tensor 37 infrared spectrophotometer with KBr disks. X-ray data were determined by an Agilent Xcalibur Nova X-ray diffractometer. NMR data were determined by a Bruker AM-400 spectrometer at 25 ºC. HPLC was used by a Shimadzu LC-20 AT equipped with an SPD-M20A PDA detector, and a YMC-pack ODS-A column (250 × 10 mm^2, S-5 μm, 12 nm) was used for semi-preparative HPLC separation. Silica gel (300–400 mesh, Qingdao Haiyang Chemical Co., Ltd.), reversed-phase C_{18} (Rp-C_{18}) silica gel (12 nm, S-50 μm, YMC Co., Ltd.), and Sephadex LH-20 gel (Amersham Biosciences) were used for column chromatography (CC). All solvents were of analytical grade (Guangzhou Chemical Reagents Company, Ltd.).
